# Reduction in Rumen Tetracycline-Insensitive Bacteria during a Grain Challenge Using the Isoflavone Biochanin A

**DOI:** 10.3390/vetsci10040273

**Published:** 2023-04-04

**Authors:** Michael D. Flythe, Brittany E. Davis, Isabelle A. Kagan

**Affiliations:** 1 Forage-Animal Production Research Unit, Agricultural Research Service, United States Department of Agriculture, Lexington, KY 40546, USA; 2 Department of Animal & Food Sciences, University of Kentucky, Lexington, KY 40546, USA

**Keywords:** antibiotic growth promoter, antibiotic-resistance, antimicrobial-resistance, functional feed, forage, legume, phytochemical, plant secondary metabolite

## Abstract

**Simple Summary:**

Biochanin A is an isoflavone, a small molecule produced by red clover (*Trifolium pratense*) and other legumes. Biochanin A promoted weight gain in growing steers in a manner similar to feed antibiotics. In laboratory experiments, biochanin A prevented bacteria from pumping out antibiotics and other inhibitory compounds, which increased their sensitivity to those inhibitors. It seemed logical that biochanin A might reduce the number of bacteria that were insensitive to an inhibitor, for example, the antibiotic tetracycline. The number of tetracycline-insensitive bacteria in the bovine rumen increases when the animals are fed high-starch diets. We fed steers (*Bos taurus*) diets with increasing concentrations of cracked corn (*Zea mays*) and enumerated the tetracycline-insensitive bacteria. Experimental groups received (1) biochanin A, (2) a well-studied feed antimicrobial, (3) no intervention, or (4) they were left on a forage-only diet. Biochanin A mitigated the increase in tetracycline-insensitive bacteria during the high-starch periods. These results support the idea that biochanin A can make some bacteria more sensitive to antibiotics and other inhibitors in animals, just as it does in the laboratory. More research is needed to determine the roles of forage legumes as alternatives to growth-promoting antibiotics and in combating the spread of antibiotic-resistant bacteria.

**Abstract:**

The isoflavone biochanin A was previously shown to promote weight gain in growing steers by selectively inhibiting rumen bacteria-like growth-promoting feed antibiotics. The hypothesis that biochanin A inhibited the action of drug efflux pumps was tested by enumerating tetracycline-insensitive bacteria from steers in a subacute rumen acidosis (SARA) challenge. Steers (*n* = 3/group) treatment groups were forage only, SARA control, SARA with monensin (0.2 g d^−1^), and SARA with biochanin A (6.0 g d^−1^). As the steers were stepped up from the forage-only basal diet to 70% cracked corn, the number of rumen bacteria enumerated on two tetracycline-containing media types (nutrient glucose agar and tetracycline, and bile esculin azide and tetracycline) increased (*p* < 0.05) from as little as 1.7(10^5^) to as great as 6.7(10^6^) cfu mL^−1^ on the nutrient glucose agar in the SARA and monensin control groups. The biochanin A group maintained the same number of tetracycline-insensitive bacteria as the forage-only controls (*p* > 0.05). The effects were similar to the more selective media type, but the differences were smaller. These results support the hypothesis that biochanin A inhibits the activity of drug efflux pumps in vivo.

## 1. Introduction

It is well-established that ionophores and some other classes of antibiotics promote weight gain and feed efficiency in ruminants when included in certain diets [1,2]. The selective inhibition of rumen microorganisms occurs when growth-promoting feed antibiotics (GPA) are consumed by ruminants. For example, tetracyclines are broadly antibacterial, while ionophores are especially effective against Gram-positive bacteria and protists [2]. This selective inhibition is beneficial in terms of both carbon efficiency and nitrogen efficiency. When the bacteria that convert amino acids into ammonia (for example, Hyper Ammonia-Producing Bacteria, HAB) are inhibited, amino acid deamination decreases, and a greater proportion of the dietary amino-nitrogen is metabolically available to the animal. Similarly, when the bacteria and protists that produce H_2_ are inhibited, less H_2_ is available for interspecies hydrogen transfer and methanogenesis [2]. Decreasing the loss of nitrogen and carbon from the system improves the growth performance of the animals. Additionally, less nitrogen and carbon are lost in the environment (in other words, ammonia in the groundwater and methane in the atmosphere), which is a measurable sustainability benefit of the use of GPA [3].

Despite the production and environmental benefits of GPA, the practice is called into question by its potential to generate antibiotic-resistant bacteria. There is evidence that GPA and even veterinary antibiotic use contribute to antibiotic resistance in bacteria associated with livestock [4,5,6]. Concerns about the spread of antibiotic-resistant bacteria have led to restrictions on the use of GPA in both Europe and the United States. It is advantageous to reduce the use of clinically important antimicrobials while expanding alternative growth-promoting technologies.

There are a variety of alternatives to both GPA and prophylactic veterinary antibiotics for both ruminants and other livestock. Plant-based alternatives to ruminant GPA include isoflavones, which are the subject of this article. Isoflavones are a class of phenolic compounds that are produced primarily by legumes and are known for their antioxidant and estrogenic properties, and more recently for their roles in cancer and cardiovascular health [7]. Several studies showed the antimicrobial activity of isoflavones against clinically relevant bacteria, such as staphylococci and mycobacteria [8,9,10,11]. In particular, work by Lechner and colleagues [9] indicated that the mechanism of antimicrobial action of some isoflavones was the potentiation of other antimicrobial compounds. The hypothesis is that isoflavones can interfere with the action of membrane-bound multi-drug efflux pumps, such as TetA. Various phenolic compounds have been found to potentiate the activity of antibiotics. For example, biochanin A and genistein potentiated the activity of berberine against *Staphylococcus aureus*, decreasing the minimum inhibitory concentration (MIC) of berberine from 500 to 31.3 µg/mL [8]. Ethyl 3,4-dihydroxybenzoate, found in wine and peanuts, decreased the IC_50_ of erythromycin against drug-resistant *E. coli* by a factor of 4 [12]. The potentiation of antibiotic activity suggests the inhibition of bacterial efflux pumps. Efflux pump inhibition has several mechanisms, one of which is competitive or noncompetitive inhibition of the efflux pump proteins [12]. Inhibition requires the ability to bind to the efflux pump protein. Having a structure similar to that of the antibiotic should facilitate binding to the same protein. The bicyclic moiety of biochanin A somewhat resembles part of the tetracycline structure as it has a carbonyl and a hydroxyl group on the bicyclic moiety. The methoxy substitution on C-4′ may contribute to the ability to potentiate tetracycline activity because Guz et al. [13] found that flavones with a methoxy group (or some other small alkyl group) on the 4′ carbon had a greater ability to potentiate berberine than flavones not substituted at that carbon or substituted with a hydroxyl group. Biochanin A is among the more hydrophobic isoflavones, being the last of the clover flavonoids to elute from a C18 HPLC column [14]. Hence, it would be expected to bind well to an interior (hydrophobic) region of a protein, such as the distal binding pocket of a drug efflux pump, where ethyl 3,4-dihydroxybenzoate has been proposed to bind [12].

The hypothesis that biochanin A inhibited efflux pumps was supported by an experiment in which *Mycobacterium smegmatis* cells were loaded with ethidium bromide [9]. When the cells were metabolically active the rate of ethidium bromide efflux from the cells could be determined by the spectrophotometric quantification of ethidium bromide in the supernatant. However, when certain isoflavones were added the rate of efflux decreased in a dose-dependent manner.

Our research group discovered the antimicrobial activity of biochanin A against a rumen bacterium using bioassay-guided fractionation [15], with evidence of the potentiation mechanism of action being shown experimentally [16]. Briefly, biochanin A was not inhibitory to a ruminal *Peptostreptococcus* species unless sterilized rumen fluid was added to the growth medium [16]. These results indicated that a sub-inhibitory compound in the rumen fluid worked in concert with biochanin A. The volatile fatty acids produced by the rumen bacteria as fermentative end products were logical candidates. A mixture of typical rumen VFA was not inhibitory when mixed with biochanin A up to 200 ppm. However, a sub-inhibitory concentration of the bacteriocin, bovicin HC5, became inhibitory when as little as 2 ppm biochanin A was added. It is plausible that biochanin A potentiated the activity of antimicrobial compounds, like bacteriocins, that are made by members of the rumen microbial community under normal conditions.

If biochanin A could effectively decrease the MIC of natural rumen metabolites, then it was a candidate alternative to GPA, which was explored [17,18,19,20]. Furthermore, if the mechanism of action is drug efflux pump inhibition, then feeding biochanin A might also reduce the number of susceptible antibiotic-resistant bacteria in vivo. It is established that the number of tetracycline-resistant bacteria in the bovine rumen increases as animals are transitioned from forage-based diets to high concentrations of cereal grains [21]. A sub-acute rumen acidosis (SARA) challenge was used to explore the impact of supplementary biochanin A or a more typical feed antimicrobial (monensin) on the viable enumeration of bacteria that can grow in the presence of tetracycline.

## 2. Materials and Methods

### 2.1. Animals and Diets

Animal husbandry and procedures were approved by the University of Kentucky Institutional Animal Care and Use Committee (protocol #: 2016–2450) and were consistent with the Guide for Care and Use of Agricultural Animals in Research and Teaching [22]. The feeding experiment was performed as described by Harlow and colleagues [23] in outdoor pens at the University of Kentucky’s C. Oran Little Research Farm, Woodford County, Kentucky, USA. In summary, the experimental animals included twelve mature rumen fistulated steers (Holstein, starting BW: 373 ± 7.5 kg). The basal diet was corn silage (45% DM, 8.39% CP, 25.75% ADF, 50.11% NDF, and 78.91% IVTD) with dried distillers’ grains + solubles (DDGS; 86% DM, 25.18% CP, 18.62% ADF, 27.65% NDF, and 84.71% IVTD) added to meet protein requirements. All animals were on the basal diet ad libitum for 14 days of adaptation before measurements. During the adaptation period, all steers had access to free-choice water and conventional loose mineral (KNS #600). All animals remained on the basal diet ad libitum for the next 7 d in the High Fiber period, in which measurements were made as indicated, and the free-choice mineral was withdrawn. During the High Fiber and subsequent periods, conventional loose mineral mixed with each treatment (biochanin A, monensin, or mineral-only), and a portion of the allotted DDGS (0.45 kg head^−1^) was transruminally administered (9:00 each day) to achieve one of four treatment groups, to which the steers were randomly assigned (*n* = 3/treatment group): (1) high fiber control (HF CON), (2) SARA control (SARA CON), SARA with monensin (MON CON), or SARA with biochanin A (BCA). HF CON and SARA CON received unamended, un-medicated loose mineral (Beef Pasture Mineral, KNS #600; Kentucky Nutrition Service, Lawrenceburg, KY, USA). MON CON received 0.200 g d^−1^ monensin (Rumensin Beef Mineral Medicated Premix, KNS #634; KNS). BCA received 6.0 g d^−1^ purified biochanin A (Indofine Chemical Co., Hillsborough, NJ, USA) in an un-medicated loose mineral. HF CON remained on the ad libitum basal diet and mineral-only treatment for the remainder of the study. The SARA CON, MON CON, and BCA assigned steers were transitioned to a high-corn diet ad libitum as follows. At the end of the 7-day High Fiber period, SARA CON, MON CON, and BCA were fed a diet of 40% cracked corn (88% DM, 9.77% CP, 19.21% ADF, 12.27% NDF, and 71.31% IVTD)/60% basal diet for 4 days and then were increased to a 70% cracked corn/30% basal diet for 4 days (as fed; [23]). The nutrient composition of the corn silage, DDGS, and cracked corn are provided in [23]. As demonstrated by pH measurements after 0, 2, 4, and 8 h in a companion study [23], rumen fluid pH was below 5.60 in steers consuming 40% cracked corn for 2 or 4 h, and below 5.60 in steers consuming 70% cracked corn at all time points in which pH was tested. These pH values indicated the induction of SARA [23].

### 2.2. Media and Culture Techniques

Phosphate buffered saline (PBS) contained, per L: 8 g NaCl, 0.2 g KCl, and 1.44 g Na_2_HPO_4_. The salts were added to deionized water and the pH was adjusted to 7.4 with HCl. The PBS was autoclaved (121 °C, 20 min), cooled under O_2_-free N_2_, dispensed anaerobically into Hungate tubes, and autoclaved for sterility.

Bile esculin azide agar (BEA) was prepared as indicated by the manufacturer (Enterococcosel™, Becton Dickinson, Franklin Lake, NJ, USA) in sterile, disposable Petri plates. When indicated, BEA was amended with filter-sterilized tetracycline solution to a final concentration of 10 µg mL^−1^ to make BEA + T.

Nutrient agar with glucose (NAG) contained, per L: 5 g Trypticase Peptone (Becton Dickinson, Franklin Lake, NJ, USA, 3 g yeast extract (Becton Dickinson, Franklin Lake, NJ, USA), 5 g NaCl, 1 g glucose, and 15 g agar (Becton Dickinson, Franklin Lake, NJ, USA). The pH was adjusted to 6.8 prior to autoclaving (121 °C, 20 min) and pouring into sterile, disposable Petri plates. When indicated, NAG was amended with filter-sterilized tetracycline solution to a final concentration of 10 µg mL^−1^ to make NAG + T.

The steers were sampled via the rumen fistula 4 h after feeding [23]. Approximately 1 kg digesta (a composite from the dorsal and ventral rumen) was removed from each individual and transported to the laboratory where it was squeezed through a muslin. The rumen fluid was serially diluted 1:10 in sterile PBS using tuberculin syringes with 18- or 21-gauge needles. Samples (200 µL) from each PBS dilution were plated onto each of the four solid media types using disposable sterile spreaders. The plates were incubated (39 °C; 96 h). Plates with 30 to 300 colonies were counted to obtain raw viable numbers. Raw viable numbers from each individual on a given sample day were averaged and normalized by a log transformation. The data were analyzed (SAS v.9.3, SAS Institute Inc., Cary, NC, USA [23]). Raw viable numbers from each animal on a given sample day were averaged and normalized by a log transformation. The data were analyzed using the MIXED procedure of SAS with repeated measures and animals as the experimental unit (SAS v.9.3, SAS Institute Inc., Cary, NC, USA). Block, sample day, treatment, and the interaction of sample day and treatment were analyzed as fixed effects and the animal was included as a random variable. The Kenward-Roger method was used to compute the denominator degrees of freedom for each fixed effect, a compound symmetry covariance structure was included in the repeated statement, and when a significant fixed effect was detected, the means were separated with the PDIFF option. Statistical significance was set at *p* < 0.05 with a trend considered at *p* < 0.1. The replication utilized in the current experiment was animal. No pseudo-replication was performed. The identification of outliers in the data was assessed using the median and interquartile deviation method (above or below the 75th or 25th percentile by a factor of 1.5 times the interquartile range). No outliers were identified. 

## 3. Results

**Figure 1:**
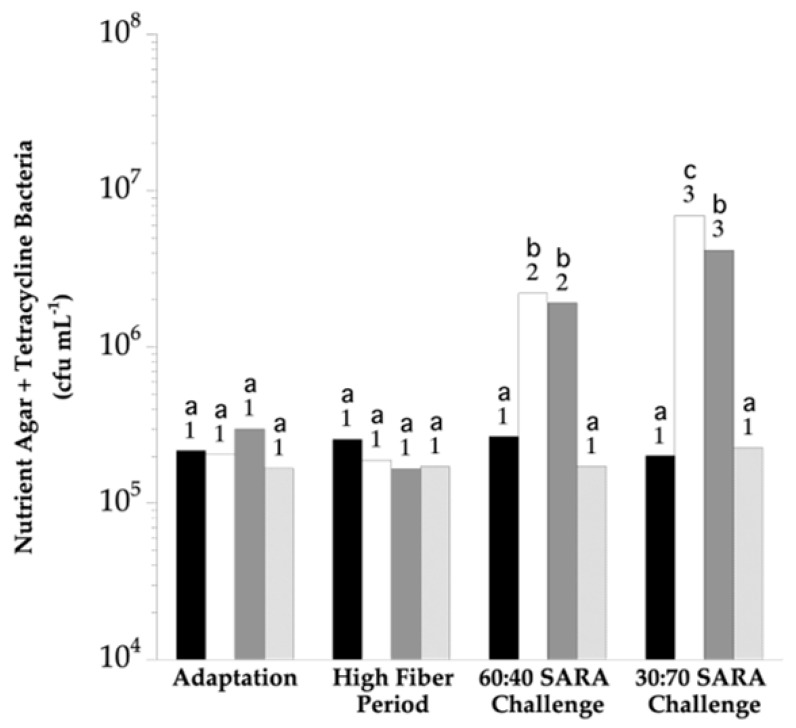
The effect of biochanin A on the viable number of tetracycline-insensitive rumen bacteria on an aerobic, non-selective media. The horizontal axis indicates the experimental period: *Adaptation*, basal diet only, no treatments; *High Fiber*, basal diet only, treatments imposed; *60:40 SARA Challenge*, 40% cracked corn, treatments continued; 30:70 SARA Challenge, 70% cracked corn, treatments continued. Bars indicate viable numbers: black, HF CON; white, SARA CON; dark grey, MON CON (monensin, 0.20 g d^−1^); light grey, BCA (biochanin A, 6.0 g d^−1^). The means lacking a common letter are different within the period (*p* < 0.05). The means lacking a common number differ over treatment periods (*p* < 0.05).

The enumeration on the Nutrient Agar Glucose Tetracycline (NAG + T) plates indicated 1.7 × 10^5^ to 2.7 × 10^5^ viable bacteria per mL rumen fluid (cfu mL^−1^) during the adaptation and high-fiber periods (Figure 1). These viable numbers were not different during the adaptation and high-fiber periods despite the imposition of treatments (*p* > 0.05). NAG + T viable numbers for the HF CON group remained similar to the high-fiber period for the remainder of the study (*p* > 0.05). NAG + T viable numbers in the rumens of SARA CON and MON CON steers increased approximately 10-fold (*p* < 0.05) when cracked corn made up 40% of the ration. The viable numbers were even greater when cracked corn made up 70% of the ration (*p* < 0.05); 6.7(10^6^) and 4.2(10^6^) cfu mL^−1^ for the SARA CON and MON CON groups, respectively. The NAG + T viable numbers in the rumens of the BCA group remained low and indistinguishable from the adaptation period, high-fiber period, and from the HF CON group, even during the corn challenge periods (*p* > 0.05).

**Figure 2:**
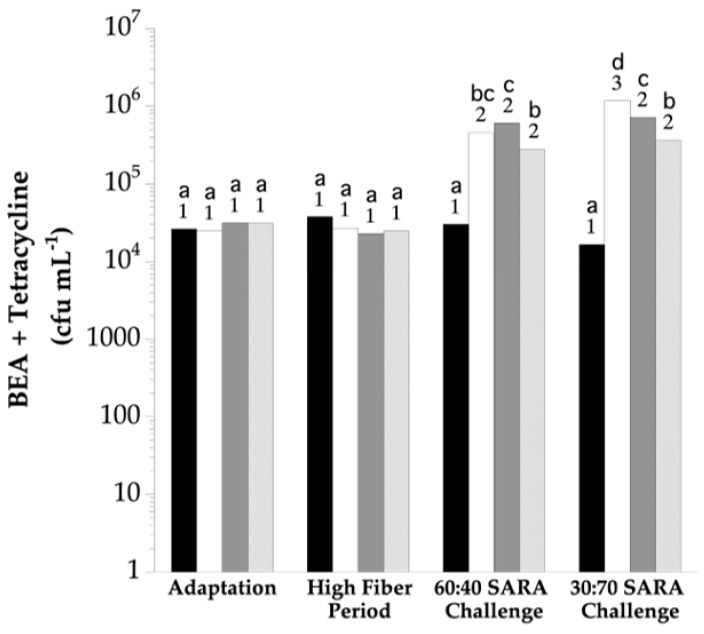
The effect of biochanin A on the viable number of tetracycline-insensitive rumen bacteria on Bile Esculin Azide Agar. The horizontal axis indicates the experimental period: *Adaptation*, basal diet only, no treatments; *High Fiber*, basal diet only, treatments imposed; *60:40 SARA Challenge*, 40% cracked corn, treatments continued; 30:70 SARA Challenge, 70% cracked corn, treatments continued. Bars indicate viable numbers: black, HF CON; white, SARA CON; dark grey, MON CON (monensin, 0.20 g d^−1^); light grey, BCA (biochanin A, 6.0 g d^−1^). The means lacking a common letter are different within the period (*p* < 0.05). Means lacking a common number differ over treatment periods (*p* < 0.05).

The enumeration on the Bile Esculin Azide + tetracycline (BEA + T) plates indicated 2.5(10^4^) to 3.8(10^4^) cfu mL^−1^ during the adaptation and high-fiber periods (Figure 2). These viable numbers were not different during the adaptation and high-fiber periods even though treatments were imposed during high-fiber (*p* > 0.05). BEA + T viable numbers for the HF CON group remained similar to the high-fiber period for the remainder of the study (*p* > 0.05). BEA + T viable numbers in the rumens of SARA CON and MON CON steers increased approximately 10-fold (*p* < 0.05) when cracked corn was 40% of the ration and increased further when corn was increased to 70% (*p* < 0.05); 6.7(10^6^) and 4.2(10^6^) cfu mL^−1^ for the SARA CON and MON CON groups, respectively. The BEA + T viable numbers in the rumens of the BCA group were indistinguishable from all others during the adaptation and high-fiber periods (*p* > 0.05). Like SARA CON and MON CON, the BCA group showed an increase in colonies on the BEA + T agar during the corn challenge periods. The viable numbers in the rumens of BCA steers were less than those in the MON CON group during the 70% cracked corn period, but only slightly so (*p* < 0.05).

## 4. Discussion

Antibiotic resistance is defined as a derived, rather than ancestral, trait [24]. In other words, a bacterial isolate is not said to be resistant to an antibiotic unless the species was previously sensitive at some point in its evolutionary history. Isolates were not identified in this study. Thus, it is more accurate to characterize the viable numbers reported here as tetracycline-insensitive, than tetracycline-resistant.

Two simple media types were selected as the enumeration media [25]. Nutrient agar is a non-selective medium that supports the growth of a variety of aerobes and facultative anaerobes. A standard composition for nutrient agar was amended with glucose to promote an even broader range of bacteria, including many well-studied coliforms, which are typically members of the Phylum Proteobacteria. The nutrient agar was also amended with tetracycline, which belongs to the tetracycline class of antibiotics, and is long considered to be broad spectrum [26]. Bile esculin azide agar is selective for Lancefield group D streptococci and enterococci [25]. This group includes *Streptococcus bovis*, which becomes a predominant rumen bacterium in starch-associated rumen acidosis [27]. Bile esculin azide agar with tetracycline was selected to specifically enumerate tetracycline-resistant members of the streptococci in anticipation of increased streptococci during the SARA challenge [23].

Auffret and colleagues [21] reported a relationship between the beef steer diet and antimicrobial-resistance genes in the rumen microbiota. In total, 32 antimicrobial-resistance genes were more abundant in concentrate-fed animals than in forage-fed animals, even though there was no difference in the antimicrobial administration. Similarly, when we added and increased cracked corn in a forage-based diet, a greater number of tetracycline-insensitive colonies were enumerated on each media type. Auffret et al. [21] also noted a greater abundance of Proteobacteria relative to Firmicutes and Bacteroides in a 16 S analysis. Phylum Proteobacteria includes Gram-negative facultative anaerobes, such as *Escherichia coli* and *Salmonella enterica* that will grow on nutrient agar. Note that neither the previous [21] nor the current study included animals receiving antibiotics.

The result that animals receiving biochanin A had fewer isolates on tetracycline-containing media is consistent with the hypothesis that isoflavones interfere with antimicrobial efflux mechanisms. These results are consistent with previous work. Lechner and colleagues discovered that biochanin A was one of several isoflavones that inhibited ethidium bromide efflux from cells [9]. Some other studies showed that flavonoid compounds potentiated the activity of other antimicrobial compounds [8,10,28]. In fact, Qu and coworkers showed that the flavone, quercetin, potentiated the activity of tetracycline against tetracycline-resistant *E. coli* [28]. Similarly, previous work by our research group indicated that biochanin A potentiated the activity of the rumen native antimicrobial, bovicin HC5 [16].

Based on the accumulated evidence, we propose that biochanin A potentiates the activity of antimicrobial compounds that are naturally produced in the rumen (e.g., bovicin) by inhibiting the efflux pumps that confer resistance to those compounds. When the antimicrobial efflux pumps are inhibited, the organisms that express them become sensitive to the antimicrobial compounds. The viable numbers of the sensitized bacteria could then be reduced by the natural antimicrobial compounds in the rumen, which might increase in high-starch diets. For example, the producer of bovicin HC5 is *S. bovis* HC5, which is an amylolytic bacterium that flourishes during rumen acidosis [16,27]. Furthermore, a drug efflux pump inhibitor would reduce the adaptive value of expressing an efflux pump for any reason. The absence of adaptive value would increase the fitness cost of expressing the efflux pump, which could decrease the frequency of genes in the population. In other words, there is no selective pressure to keep a gene that does not enhance survival.

Antibiotic resistance is a public health problem. The connection between veterinary medicine and public health is clear in the context of zoonotic disease. However, the improvement of public health outcomes is not a role that we traditionally associate with the field of ruminant nutrition [29]. Conversely, few would question that public health and human nutrition are inseparable. A useful concept from human nutrition is that of functional foods, which are sometimes called food-as-medicine but can be more broadly defined as foods that have benefits beyond simple nutrition. Clovers and other forage legumes could be thought of as functional feeds. It is noteworthy that the dietary level of biochanin A used in this study could be achieved by feeding red clover [23]. Other benefits of red clover isoflavones (biochanin A, formononetin, etc.) include inhibiting starch fermentation [30], decreasing amino acid deamination [15,16,17], promoting weight gain on forage-based diets [17,19], and improving blood flow during fescue toxicosis [31,32]. These benefits can be achieved in each case without extracting the isoflavones and giving them as a drug. The animal can be fed the legume or simply graze it.

## 5. Conclusions

Fewer tetracycline-insensitive rumen bacteria were enumerated from steers given biochanin A during the final period of the SARA challenge than from steers given monensin, or from challenged steers receiving neither biochanin A nor monensin. The results support the hypothesis that biochanin A potentiates the antimicrobial activity of other compounds and that they are consistent with other reports of natural products potentiating the activity of antibiotics. It is plausible that dietary biochanin A can decrease the fitness value of drug efflux pumps among rumen bacteria thereby decreasing the frequency of the bacteria that require them. The results suggest another beneficial property of biochanin A for ruminants. More importantly, the results illustrate the complex roles of phytochemicals in the gastrointestinal microbiology of herbivores.

## Data Availability

Not applicable.
